# A review of combined functional neuroimaging and motion capture for motor rehabilitation

**DOI:** 10.1186/s12984-023-01294-6

**Published:** 2024-01-03

**Authors:** Emanuel A. Lorenz, Xiaomeng Su, Nina Skjæret-Maroni

**Affiliations:** 1 Department of Computer Science, Norwegian University of Science and Technology, Trondheim, Norway; 2 Department of Neuromedicine and Movement Science, Norwegian University of Science and Technology, Trondheim, Norway

**Keywords:** Multimodal, Neuroimaging, Motion capture, Motor function, Rehabilitation, Assessment, Review

## Abstract

**Background:**

Technological advancements in functional neuroimaging and motion capture have led to the development of novel methods that facilitate the diagnosis and rehabilitation of motor deficits. These advancements allow for the synchronous acquisition and analysis of complex signal streams of neurophysiological data (e.g., EEG, fNIRS) and behavioral data (e.g., motion capture). The fusion of those data streams has the potential to provide new insights into cortical mechanisms during movement, guide the development of rehabilitation practices, and become a tool for assessment and therapy in neurorehabilitation.

**Research objective:**

This paper aims to review the existing literature on the combined use of motion capture and functional neuroimaging in motor rehabilitation. The objective is to understand the diversity and maturity of technological solutions employed and explore the clinical advantages of this multimodal approach.

**Methods:**

This paper reviews literature related to the combined use of functional neuroimaging and motion capture for motor rehabilitation following the PRISMA guidelines. Besides study and participant characteristics, technological aspects of the used systems, signal processing methods, and the nature of multimodal feature synchronization and fusion were extracted.

**Results:**

Out of 908 publications, 19 were included in the final review. Basic or translation studies were mainly represented and based predominantly on healthy participants or stroke patients. EEG and mechanical motion capture technologies were most used for biomechanical data acquisition, and their subsequent processing is based mainly on traditional methods. The system synchronization techniques at large were underreported. The fusion of multimodal features mainly supported the identification of movement-related cortical activity, and statistical methods were occasionally employed to examine cortico-kinematic relationships.

**Conclusion:**

The fusion of motion capture and functional neuroimaging might offer advantages for motor rehabilitation in the future. Besides facilitating the assessment of cognitive processes in real-world settings, it could also improve rehabilitative devices’ usability in clinical environments. Further, by better understanding cortico-peripheral coupling, new neuro-rehabilitation methods can be developed, such as personalized proprioceptive training. However, further research is needed to advance our knowledge of cortical-peripheral coupling, evaluate the validity and reliability of multimodal parameters, and enhance user-friendly technologies for clinical adaptation.

## Introduction

Technological advancements have paved the way for innovative neurorehabilitation methods. State-of-the-art sensor systems now enable the minimal intrusive acquisition of various biological parameters in a wide range of settings. Additionally, increased computational power allows for more complex analysis methods and accelerated processing [1–4]. These advancements greatly benefited the field of Mobile Brain/Body Imaging (MoBi), which focuses on the acquisition and analysis of complex multimodal signal streams, such as neurophysiological data (e.g., electroencephalography and functional near-infrared spectroscopy) and behavioral data (e.g., motion capture and eye-tracking). This has led to new insights into the brain’s cortical mechanisms during ecological valid movement and is thought to not only guide the development of new rehabilitation practices but also become a future tool for assessment and therapy in neurorehabilitation [5–7].

### Sensor-based practices in motor rehabilitation

Acquired neurological disorders, such as stroke, are the leading cause of long-term disability worldwide. Consequently, it is crucial to develop improved methods to elevate the quality of life for individuals impacted by these disorders. [8]. Motor rehabilitation focuses on improving functionality by promoting internal processes that drive spontaneous neural restoration via neuroplastic changes [9, 10]. Current practices involve clinicians manually assessing and treating sensory-motor deficits by facilitating motor learning, but these methods are resource-intensive, and the efficacy is often debated [11–13]. Utilizing validated sensor data can help objectively evaluate those existing rehabilitation methods while also introducing new assessments and therapeutic methods. A more comprehensive and robust understanding of a patient’s functional deficits and training effects can be achieved by concurrently examining movement biomechanics and underlying neuroplastic changes. An additional benefit of incorporating MoBi into motor rehabilitation is the ability to evaluate patient progress within an ecologically valid context, as the training mirrors relevant and task-specific movements in real or virtual environments [6, 7]. This approach aligns with the nature of activities of daily living (ADLs), characterized by task-, environment-specific, and complex movements, and has been demonstrated to yield the highest benefit for functional recovery [13, 14].

### Motion capture technologies for biomechanical assessments

Regaining motor function is a top priority in rehabilitating after acquired neurological disorders like stroke or traumatic brain injuries (TBI) [15]. Traditional motor rehabilitation primarily focuses on continuously analyzing movement biomechanics and providing feedback on the quality. During the initial physical examination, these biomechanical assessments are crucial for selecting the most effective treatment strategy and determining a probable prognosis [16]. In the subsequent rehabilitation, biomechanical measures assist in building muscle strength and mobility by evaluating functional changes and providing corrective feedback. While manual assessment scores, like the Fugl-Meyer Assessment (FMA), have traditionally been used for this purpose, various motion capture technologies have been applied to assess relevant movement biomechanics automatically [17].

Optical motion capture devices are a common clinical method for capturing human motion. These systems utilize camera systems to either capture markers attached to the patient or estimate joint positions directly from video data using machine learning. Because of their high spatial and temporal precision, the aforementioned marker-based systems are still considered the gold standard. However, their resource-intensive set-up often limits their use in clinical settings [1, 18]. In recent years, markerless motion capture systems have advanced significantly, particularly with regard to spatial accuracy and usability [19].

In addition to optical sensors, mechanical and magnetic sensor systems are also used for motion capture. Both systems require the manual application of sensor systems on the joint or limb of interest, making global motion tracking of multiple joints impractical [2]. However, unlike optical motion tracking systems, mechanical and magnetic sensors do not require a line of sight between the marker/joint and a camera system, thus avoiding the issue of occlusion [18].

### Functional neuroimaging technologies for neurological assessment

Aside from movement mechanics, the underlying cortical and subcortical processes are consulted in neurorehabilitation. This allows for directly assessing, predicting, and addressing altered cortical activity related to brain disorders and subsequent changes during therapy [20, 21]. Recent studies highlight the benefits of employing functional neuroimaging for treating neuromotor diseases, such as using brain-computer interfaces (BCI) for motor training [22].

Portable and non-invasive methods, such as electroencephalography (EEG) and functional near-infrared spectroscopy (fNIRS), are increasingly used in motor rehabilitation due to their relative simplicity and affordability. The electric activity measured by the EEG is based on the sum of inhibitory and excitatory postsynaptic potentials of large neuronal networks firing synchronously in response to internal or external stimulus [23]. Conversely, fNIRS employs near-infrared light to detect changes in cortical hemodynamic activity resulting from earlier changes in neural activity [24]. Compared to fNIRS, EEG offers a higher temporal resolution (EEG: < 1 ms; fNIRS: < 100 ms), which is advantageous for generating real-time feedback on movement-related changes in rehabilitation applications. The downside is that EEG is more susceptible to environmental (e.g., power line noise) and physiological (e.g., movement, eye-blink, sweat) artifacts, necessitating extensive pre-processing before feature extraction to obtain valid results [25, 26].

Comparably offer non-portable technologies, like Magnetoencephalography (MEG), functional magnetic resonance imaging (fMRI), and positron emission tomography (PET), superior spatial resolution (MEG: 2–3 mm, fMRI: 1 mm, PET: 2.5 mm). However, their applicability in motor rehabilitation is limited due to their static nature and high cost, necessitating magnetically shielded rooms (MEG, fMRI) or complex logistics (PET) [27–29]. MEG, similar to EEG in temporal accuracy, records changes in the brain’s less distorted magnetic fields using magnetometers, providing higher spatial resolution (2–3 mm) than EEG (7–10 mm). Notably, portable MEG systems are in development [27, 30, 31]. fMRI, similar to fNIRS in temporal accuracy, detects hemodynamic changes by monitoring the electromagnetic signals emitted by hydrogen protons during imposed magnetic moment (spin) changes. Unlike fNIRS, fMRI offers high spatial accuracy throughout brain, not limited predominantly to the cortex [28]. PET, beyond its non-portability, is invasive, requiring radioactive tracer injection. External detectors track radioisotope metabolization and distribution, providing brief insights into specific processes with high spatial but low temporal resolution (1 min) [29].

### Multimodal measurements in motor rehabilitation

Research on multimodal assessments in the field of neurorehabilitation is generally limited. Nevertheless, recent reviews investigated the fusion of functional neuroimaging and electromyography (EMG), as well as biomechanical measurements and EMG. [16, 32, 33]. Combined EMG/EEG measures, such as corticomuscular coherence (CMC) representing corticospinal synchronization, correlate with motor improvement. However, their reliability is insufficient for the clinical assessment of functional deficits [16, 34]. Hybrid BCIs for motor rehabilitation increasingly apply combined EMG/EEG to improve the robustness of neural decoding clinical environments [33, 35].

On the other hand, the fusion of kinematic/kinetic and neurological measures for motor rehabilitation remains underexplored. Despite the increase of motion capture and functional neuroimaging as separate tools for motor rehabilitation, their combined applications, which appear complementary, have received little attention outside of MoBi research on healthy participants [5–7]. Although motion capture offers insights into movement quality, functional neuroimaging focuses on cortical activity related to motor planning, initiation, execution, attention, and neural reorganization [16, 21]. Compared to traditional assessment and rehabilitation methods, combining both modalities provides a more holistic insight by covering movement from planning to execution and control, emphasizing afferent somatosensory processing and highlighting maladaptive neuroplastic changes [6, 16, 34]. Furthermore, by relying on multiple data streams, more robust rehabilitation tools can be developed that facilitate usability and improve the efficacy and validity of motor assessment and the training of complex real-life movements in ecologically valid environments [16, 34].

### Research objective

To address the current literature gap, a comprehensive review of the emerging field that combines motion capture and functional neuroimaging for motor rehabilitation is crucial. More specifically, it would be interesting to understand the diversity and maturity of the employed technological solutions as well as the reported clinical advantages of employing such a multimodal approach. Specifically, it is essential to gain insights into the diversity and maturity of the technological solutions related to this specific multimodal approach that are relevant for future clinical applications. While acknowledging that certain studies may involve multiple additional modalities, we have intentionally focused on these chosen modalities to maintain scope and specificity. Thus, this paper aims to present and discuss various studies that have examined the combined use of functional neuroimaging and motion capture in motor rehabilitation. In particular, we will elaborate on the following Research Questions (RQ):

- (RQ1): Which technologies were used to acquire combined neurophysiological and kinetic/kinematic signals?
- (RQ2): What signal analysis techniques were used?
- (RQ3): How were the modalities combined?
- (RQ4): What was the outcome of the assessment or intervention methods?
- (RQ5): Were any suggestions made on future clinical applications?

A critical evaluation of current technical approaches and features is conducted based on the descriptive analysis of the results. Following that, broader trends and recommendations for future research and development are established by incorporating supplementary literature.

## Materials and methods

### Search strategy and selection criteria

This narrative review evaluates studies published on the combined use of functional neuroimaging and motion capture in motor rehabilitation. It was conducted following an adapted version of the Preferred Reporting Items for Systematic Reviews and Meta-Analyses (PRISMA) using the platform Colandr [36, 37]. All articles that are part of this review were obtained by searching the databases of IEEE, PubMed, and Scopus on the 17th of January 2022 (updated on the 9th of May 2023) using the following search query:

((EEG) OR (Electroencephalography) OR (fNIRS) OR (functional near-infrared spectroscopy) OR (BCI) OR (Brain-Computer-Interface)) AND ((Mocap) OR (Pose Estimation) OR (IMU) OR (Inertial Measurement Unit) OR (Time-of-flight) OR (Kinect) OR (Kinematic*) OR (Movement*) OR (Motion*) AND (Rehabilitation) AND((Acquired brain injur*) OR (ABI) OR (Stroke) OR (Traumatic brain injur*) OR (TBI))  Further, all included studies had to meet the following criteria to be included:

- Article: Must be written in English and be a peer-reviewed full-length study (min. four pages).
- Rehabilitation: The study must have direct or indirect implications on rehabilitating acquired brain injuries.
- Functional neuroimaging: Only EEG and fNIRS-based systems are considered for functional neuroimaging methods due to their applicability in clinical practices.
- Motion capture: Only systems that directly measure kinetic or kinematic modalities, such as motion capture systems, are included.
- Technology: The signal from both modalities is acquired simultaneously and linked. Further, no modality other than the described functional neuroimaging and motion capture methods is used as a main modality.

### Selection process

**Fig. 1:**
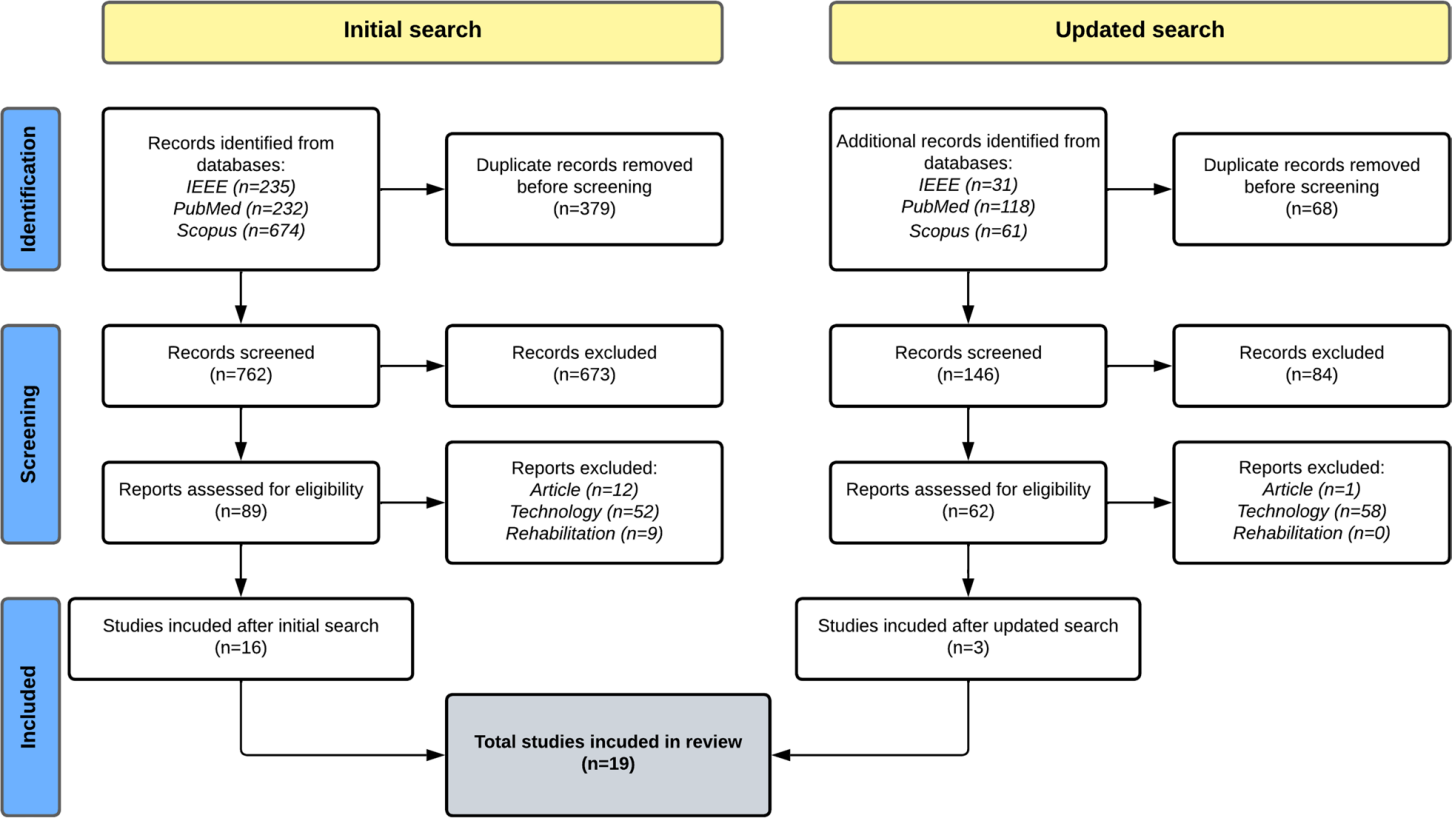
PRISMA flow diagram. Reports were excluded when they did not fulfill the stated inclusion criteria on articles (Article), the used technology and its synchronization (Technology), and its context in rehabilitation (Rehabilitation)

In Fig. 1, the PRISMA flow diagram summarizes the article selection process in detail. First, the records were extracted from the databases based on their keywords. After removing duplicates, 762 records were screened based on their titles and abstracts. A full-text screening was conducted on the remaining 89 articles, of which 16 were included in the final data extraction. During the full-text screening, publications that were prestudies of other included studies were excluded. In the updated search, 210 records were identified publications, and 62 were eligible for full-text screening, leading to 3 articles for the final data extraction. Thus, a total of 19 studies were included in the final review.

### Data extraction

Table 1 shows which data was systematically extracted and subsequently evaluated from the included studies. The review focuses on technological aspects but also includes medical use cases. Only parameters directly related to the fusion of both modalities were included in extracting information on functional neuroimaging and motion capture data acquisition and processing.

**Table 1:** Data extraction labels. A summary of the data extracted from the included studies

Study	Participants	Technology	Medical
Type	Target pathology	Neuroimaging hardware	Anatomical target
Aim	Target demographics	Neuroimaging signal processing	Application
Outcome	Control pathology	Motion capture hardware	Modality fusion
	Control demographics	Motion capture signal processing Synchronisation	

Throughout the review, we utilized various classifications to summarize the results efficiently. We provide an overview of the categories used for summarizing research types, motion capture metrics, functional neuroimaging feature domains (according to [38]), and multimodal fusion techniques in Table 2.

**Table 2:** Review categories. An explanation of the used category to summarize various extracted features

Topic	Category	Description
Research type	Basic research	Seeking new insights into cortical and biomechanical processes
	Translational research	Applying new knowledge and methods to first practical applications
	Clinical research	Evaluate the effectiveness of implemented interventions
Motion capture	Event markers	Denote specific instances in time when biomechanical changes occur
	Kinematics	Linear or angular changes within a spatial-temporal context
	Kinetics	Forces that are generated by or acting on the body
	Parameters	Physical measurements of motion
	Assessment scores	Complex features, based on single or multiple physical motion measurements
Functional neuroimaging	Time	Temporal signal changes
	Frequency	Presence and power of frequency components
	Time-Frequency	Temporal changes in the power of frequency components
	Non-linear	Non-linear analysis methods
Multimodal fusion technique	Movement event detection	Movement events to segment and classify the neural signal
	Decoder training	Multimodal training and evaluation of neural decoders
	Statistical relationship	Multimodal relationship based on statistical methods
	Parallel applications	Indirectly related biomechanical and cortical features, fused for feedback

## Results

### Study characteristics

This chapter grouped the studies according to their study characteristics, such as application type, research type, frequency of measurement, and the type of control group used. Further, a summary of the respective study aims and the temporal distribution of the publications is given. The study characteristics are summarized in Table 3.

**Table 3:** Study characteristics. The table gives an overview of the targeted application type, the research type, the frequency of data collection, what control was used, and a summary of the aim of the study. ‘–’ indicates that this does not apply to the respective study

Application	Type	Frequency	Control	Study aim	References
Diagnostic	Basic	Cross-sectional	None	Identification of submovement signatures in EEG during a double-step target displacement task	[54]
				Investigation of the effects of cognitive and motor dual tasking on gait performance and brain activities after stroke	[56]
				Investigate the participation of midfrontal theta dynamics in a behavioral monitoring system for reactive balance responses	[57]
			Healthy	Identification of particular impairments by pre- and post-movement changes in EEG after stroke	[39]
	Translational	Cross-sectional	None	Feasibility of recording kinematic and EEG data during visuomotor coordination task	[53]
				Feasibility of the combined detection of EEG and gait events during treadmill walking for rehabilitation	[55]
		Longitudinal	Healthy	Development of a multivariate analysis method to couple clinical evaluations with multimodal instrumental evaluations	[52]
Therapy	Basic	Cross-sectional	None	Evaluation of changes in cortical involvement during treadmill walking with and without BCI control of an avatar	[48]
			Healthy	Investigation of the inter-limb coordination based on brain activity and kinematic features	[42]
	Translational	Cross-sectional	None	Comparison of non-adaptive and adaptive approaches in MRCP detection for motor rehabilitation	[47]
				Investigation of a transfer learning framework for personalized decoding of TES-assisted 3D reaching task	[49]
				Development of a real-time EEG-signal processing and classification pipeline of movement intention for clinical motor rehabilitation	[51]
			Healthy	Evaluation of an active robotic upper limb exoskeleton based on gaze tracking and BCI to assist with upper limb movements	[43]
				Evaluation of movement task with visuomotor feedback based on related changes in the motor cortex	[50]
		–	–	Presentation of an exergame based on EEG and Kinect for lower-limb rehabilitation	[45]
	Clinical	Longitudinal	None	Feasibility of decoding gait kinematics during robot-assisted gait training from stroke patients using a powered exoskeleton	[44]
				Evaluation of a BCI system to assist with upper-limb functional movement rehabilitation	[40]
			Healthy	Evaluation of an assessment system for functional upper limb assessment, based on EEG and kinematic, dynamic data during planar reaching movements	[41]
			Non-Healthy	Evaluation of a novel multimodal upper-limb stroke rehabilitation exergame	[46]

**Fig. 2:**
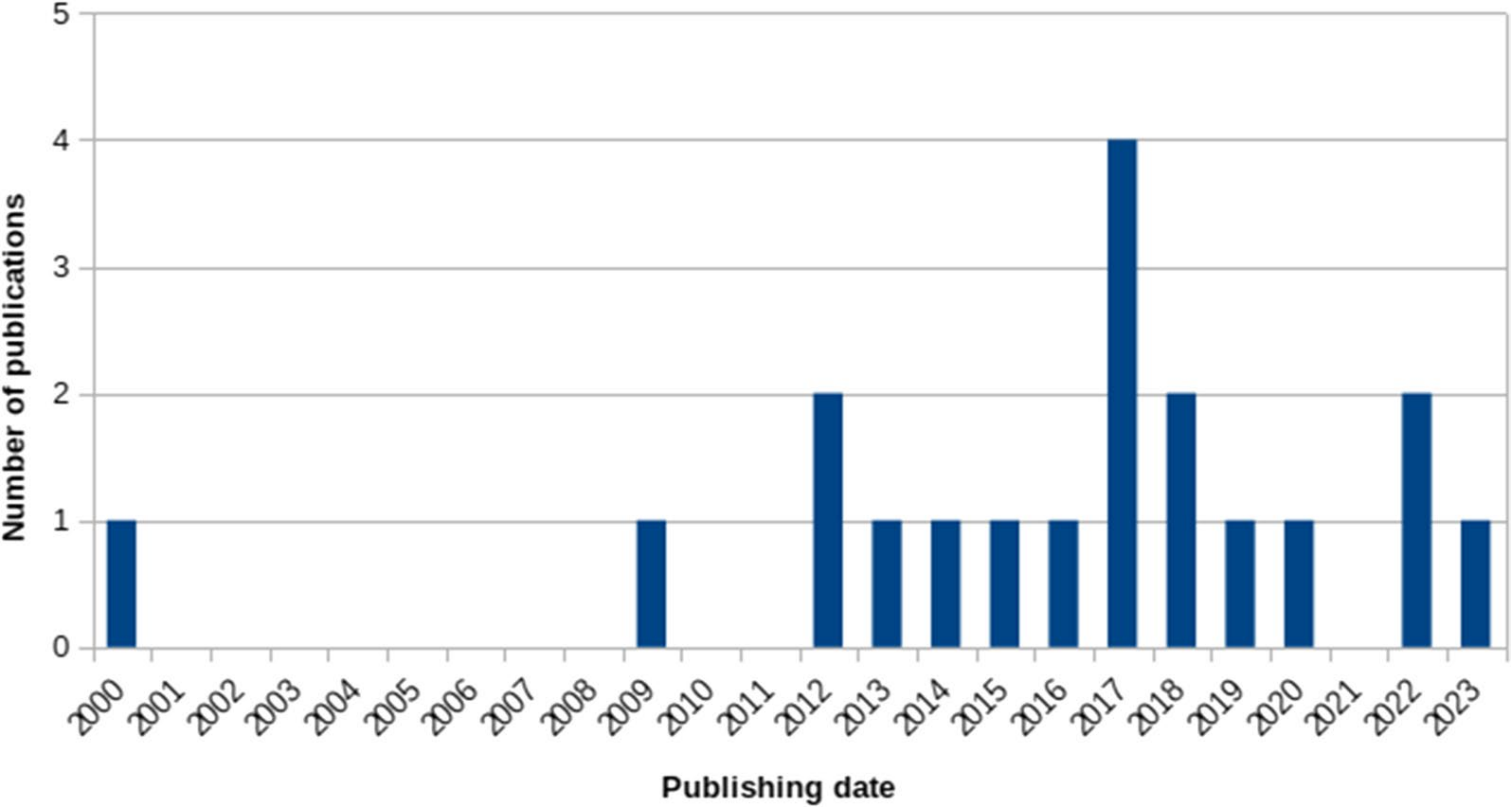
Publication chart. The number of included publications between 2000 and 2022 are displayed

The median publication date for all included studies is in 2017, with the earliest publication being from 2000 [39] (Fig. 2).

The majority of the studies (12 out of 19) focused on the development and evaluation of therapeutic applications [40–51], while the rest focused on the exploration of novel diagnostic applications [39, 52–57].

Six out of the 19 studies were categorized as basic research [39, 42, 48, 54, 56, 57], 9 out of 16 studies were categorized as translational research [43, 45, 47, 49, 50, 52, 53, 55, 58] and only four of the 16 studies could be classified as clinical research [40, 41, 44, 46]. No clinical studies were found for diagnostic applications.

Most studies were cross-sectional (13 out of 19) [39, 42, 43, 47–51, 53–57], with only one longitudinal study for translational diagnostic applications [52]. For therapeutic applications, all clinical studies were longitudinal studies [40, 41, 44, 46].

Six out of 19 studies used control groups, of which six used healthy controls without any relevant pathology [39, 41–43, 50, 52] and one using a non-healthy control group with stroke [46]. One study solely demonstrated the technical design of an intervention without including any participants [45].

### Participant characteristics

This chapter briefly summarizes the total number of involved participants (N) and the number of female participants (f), their age (Age), time since brain injury (Post-Injury), the severity of their motor disability (Motor Severity), and if the participants received any other intervention throughout the study of the involved participants.

On average, 11 (SD: 9) participants were included in the studies, with one study not including any participants, as the sole focus was on the technical design of the application [45]. Wasaka et al. [50] included with 27 participants the most.

Only 28% (SD: 29%) on average of all included participants were female, with four studies having no female participants at all [40, 44, 47, 49, 51]. The included participants’ ages differed between younger healthy (age range: 19–58) and older non-healthy (age range: 49–83) participants. In two studies, the age of the participants was not mentioned [42, 55].

Eight out of 19 studies included only healthy participants without any relevant pathological conditions [47–49, 51, 53–55, 57], of which one study had a healthy participant simulating a pathological gait pattern typical for stroke [55]. Three studies included non-healthy participants only [40, 44, 46] and the remaining six included studies had both healthy and non-healthy participants [39, 41–43, 50, 52]. All non-healthy participants were stroke patients with various aetiologies and severities and at different stages post-stroke. The time post-stroke for the included stroke patients ranged from the sub-acute phase (3 weeks) to the chronic phase (5 years).

The studies applied various motor assessment scales to quantify the participants’ functional state. For a better overview, only the predominant motor disability is summarized. Most included stroke participants showed mild functional deficits [40, 41, 44, 46, 56]. One study included stroke participants with mild to moderate deficits [39], one study participants with moderate deficits [52], and one study participants with mild to severe participants [50]. Frisoli et al. [43] did not state the motor severity of the included stroke patients.

Five out of 19 studies with non-healthy participants mentioned that some [40] or all participants [39, 41, 46, 52] underwent other rehabilitation programs.

### Anatomical targets and movements

The anatomical target of the presented studies was categorized by their general region, the targeted body part, and the investigated movement. An overview is given in Table 4. The majority of the studies focused on upper limb movements (12 out of 19) [39–41, 43, 46, 46, 49, 50, 52–54, 58], with reaching being the most commonly studied movement (7 out of 19) [40, 41, 43, 46, 49, 51, 52]. The remaining studies focused on whole-body movements, including a balance task [45, 57] and gait tasks based on overground walking [44, 56] or treadmill walking [42, 48, 55].

**Table 4:** Anatomical targets. Anatomical target and investigated the movement of included studies

Category	Anatomical target	Movement	References
Upper limb	Arm	Reaching	[40, 41, 43, 46, 49, 51, 52]
		Free/reaching/circular	[53]
	Wrist	Flexion/extension and radial/ulnar deviation	[54]
		Pronation/supination	[46]
	Finger	Triangular trajectory	[39]
	Hand	Isometric grip	[50]
Lower limb	Ankle	Isometric dorsiflexion	[47]
	Gait	Free overground walking	[44]
		Straight overground walking	[56]
		Treadmill walking	[48, 55]
		Supported treadmill walking	[42]
Whole body	Balance	Leaning	[45, 57]

### Motion capture

The motion capture systems were analyzed based on their hardware setup and the subsequent signal processing steps. Information on the hardware includes the underlying sensor category, the extent of captured joints and limbs (target), body parts from which data was acquired, the used motion capture technology, and the sample rate used for acquisition. The signal processing of the acquired signal was divided into the feature categories, used filter, the eventual use of an artifact rejection or correction method, and the final extraction of the kinematic or kinetic features. An overview of both used hardware and signal processing methods is given in Table 5.

**Table 5:** Motion capture systems. Motion Capture hardware and related signal processing and analysis. ‘–’ indicates that the authors gave no information on the respective matter

Hardware					Signal processing			References
Category	Category	Captured body part	Technology	Sample rate	Filter	Feature extraction	Features	
Mechanical	Movement event marker	Ankle	Force transducer	2048 Hz	Low-pass (3 Hz)	Force change	Movement onset	[47]
		Feet (Gait)	Pressure sensing treadmill system	20 Hz	–	–	Toe-off Heel strike	[55]
		Distal third of forearm, middle of arm	Gyroscopes	100 Hz	Low-pass (10 Hz)	Angular velocity change	Movement onset	[40]
		Hand	Force transducer	500 Hz	–	Force increase	Movement onset	[50]
	Movement event marker, Kinematic parameters/assessment	Arm via end-effector	Active robotic system	–	–	Velocity change detection	Movement onset/offset Mean velocity, Movement accuracy Movement smoothness Spectral arc-length metric Distance at robotic assist Robot assistance frequency Explored workspace area Completion time	[52]
	Kinematic parameters	Hip, knee, ankle	Accelerometer Goniometer	100 Hz	Band-pass (0–3 Hz)	–	Joint angles	[48]
		Wrist, forarm via end-effector	Accelerometer	20 Hz	–	Kalman filter	Tilt angle Acceleration Angular velocity	[46]
		Wrist	Active robotic system	1000 Hz	Low-pass (12 Hz)	(1) Euclidian norm of velocity; (2) Greedy algorithm	(1) Velocity; (2) Submovement on/offset	[54]
		Index finger	Accelerometer	500 Hz	–	–	Maximal acceleration	[39]
	Kinematic parameters/assessment Kinetic parameters	Arm via end-effector	Passive robotic system	–	–	–	Movement efficiency Number of velocity peaks Mean force vector	[41]
	Kinetic parameters/assessment	Feet (Gait)	Pressure sensing walkway system	–	–	–	Speed, Cadence, Stride time Stride length Spatio-temporal asymmetry	[56]
Mechanical, optical	Mec: Movement event marker Opt: Kinematic parameters	Mec: Feet Opt: Full body	Mec: Force plate Opt: Passive marker-based motion capture	Mec: 2000 Hz Opt: 100 Hz	Mec: - Opt: Low-pass (10 Hz)	Mec: Leg weight unloading Opt: Labeling, gap filling Marker movement	Mec: Stepping behavior by foot-offset Opt: Leaning direction	[57]
Mechanical, magnetic	Kinematic parameters	Lower body	Active robotic system	100 Hz	Low-pass (3 Hz)	–	Joint angular position Velocity	[44]
Magnetic	Movement event marker	Arm	Passive robotic system	1000 Hz	–	Joint rotation change of end effector	Movement onset	[53]
Optical	Movement event marker	Thigh, shank, foot	Active marker-based motion capture	140 Hz	–	–	Toe-off Heel strike	[42]
		Hand	Marker-less hand tracking system	100 Hz	–	Movement away from start position	Movement onset	[51]
	Kinematic parameters	Upper body	Marker-less motion capture	30 Hz	–	–	3D joint center position	[43]
		Upper body	Marker-less motion capture	30 Hz	–	–	3D joint center position	[45]
	Kinematic assessment	Arm	Active marker-based motion capture	960 Hz	–	Normalized averaged rectified jerk	Jerk	[49]

#### Motion capture hardware

Most sensor systems used for motion capture were mechanical sensors that register movement kinematics based on positional changes of the sensor itself (e.g., accelerometer [39, 46, 48] or gyroscope [40]) or positional changes of attached to a body part in reference to another (e.g., goniometer [48] or exoskeletons [41, 52, 54]). Movement kinetics were acquired via a force transducer [47, 50] or by pressure sensors [55–57]. Regarding the exoskeletons studies, only Contreras-Vidal et al. [44] reported the integrated sensor types used for kinematic and kinetic measurements.

Magnetic motion capture systems, such as those used by [44, 53], use magnetic sensors that measure movement kinematics based on changes in the surrounding magnetic field. Both studies used Hall sensors within the exoskeletons that register changes in distance to a magnet. It was not reported which sensor types any of the other exoskeletons used.

Optical motion tracking was either based on markerless motion capture systems [43, 45, 51], active marker-based motion capture system [42, 49] or passive marker-based motion capture [57]. One of the markerless systems employed was the Kinect sensor system [43, 45]. Using a pre-trained randomized decision forest, it infers the three-dimensional position of 14 joint centers based on the input of an RGB camera and a TOF camera (depth camera) [59]. Another markerless motion capture system used was the Leap Motion Orion [51]. Besides the Kinect, it is based on stereoscopic IR cameras, which feed undisclosed algorithms to interfere with the hand position in three-dimension [60]. Active and passive marker-based motion capture systems rely on correctly applying light-emitting or light-reflecting markers, which are then registered by multiple specialized cameras. Based on the extracted 2D marker position, the 3D marker positions can be triangulated. It should be mentioned that Stokkermans et al. [57] used both mechanical and optical motion capture.

Four papers did not report on the sampling rate used [41, 45, 52, 56]. However, one of those used a Kinect, which has a fixed sample rate of approximately 30 Hz [45]. Three additional studies recorded movement data at sample rates below 100 Hz [43, 46, 55]. Most studies (9 out of 19) used samples rates between 100 and 1000 Hz [39, 40, 42, 44, 48–51, 57] and three studies even sample rates above 1000 Hz up to 2048 Hz [47, 53, 54]. The systems with high sample frequency were based on magnetic and mechanical sensor systems.

#### Motion capture data analysis

For most studies (11 out of 16), no signal-preprocessing steps were reported [39, 41, 43, 45, 46, 49, 52, 53, 55, 56]. Five studies utilizing high to mid-high sampling rates applied a low-pass filter at cut-off frequencies around 12 Hz [54], 10 Hz [40], and 3 Hz [44, 47, 48], to retrain the signal component of slow movements and reject artifacts in higher frequencies. Only one study reported the manual correction of artifacts [40].

Regarding feature complexity, movement event detection has relatively low requirements for signal processing. 9 out of 19 studies used either movement onset and offset as an event marker for subsequent EEG segmentation, like movement [40, 42, 47, 50–53, 55, 57] or submovement onset/offset [54]. Such movement events were used for gait event detection, like heel strike and toe-off [42, 55], or perturbed balance-related changes in leaning direction and stepping behavior [57]. Movement onset was either defined as a change in force [47, 50], velocity [40, 54], position [51] or joint rotation [53] above a defined threshold, like a change above three standard deviations from baseline [47], change greater than 5% of the peak amplitude value [40] or extracted using a greedy algorithm [54].

Kinematic parameters, like position change [43–46, 48], movement velocity [41, 44, 46, 52, 54, 56] and acceleration [39, 46] were extracted for further multimodal analysis in eight studies. One study extracted the mean force vector as a kinetic feature [41]. In a few studies, the kinematic and kinetic parameters were not used directly but were further processed to allow for clinical movement assessment. Commonly used features of this category were measurements of movement accuracy [41, 52], movement smoothness [41, 49, 52]. Mazzoleni et al. [41] further extracted movement efficiency, Pierella et al. [52] spectral arc-length metric, time to complete tasks, and features related to robotic assistance during the task execution and Liu et al. [56] gait-specific features like cadence and stride time, length and asymmetry.

### Functional neuroimaging

The utilized functional neuroimaging hardware and signal processing methods were analyzed by extracting information on the underlying sensor technology, sensor type, number, placement, and the system’s sample rate. The signal processing of the acquired signal was divided into the feature categories, used filter, re-referencing method, signal segmentation, artifact rejection or correction methods, and the final extraction of relevant neural features. An overview of both the used hardware and signal processing methods is given in Table 6.

**Table 6:** Functional neuroimaging systems. Neuroimaging hardware and related signal processing and analysis. ‘–’ indicates that the authors gave no information on the respective matter

Hardware					Signal processing						References
Technology	Sensor number	Sensor position	Sensor type	Sample rate	Feature domain	Filter	Segmentation	Artifact correction/rejection	Feature extraction	Features	
EEG	3	Prefrontal	Passive dry	–	–	–	–	–	–	Attention level	[45]
		Central	Dry foam	512 Hz	Frequency	–	–	–	–	Theta/Beta ratio	[46]
	≤ 16	Frontal, central	Active	256 Hz	Frequency	–	Moving window (1 s)	–	CPS for 8–24 Hz SVM classification	Rest/movement intention maximum joint jerk Kinematics	[43]
			–	500 Hz	Time	Band-pass [0.15–20 Hz]	Movement onset [− 2.5 s, 1 s]	Statistical trial rejection Baseline correction Inclusion of C3 or C4	–	MRCP	[50]
		Whole	Active wet	–	(1) Time; (2) Time-frequency	Band-pass [0.5–40 Hz]	Movment onset [− 5 s, 1 s]	1,2) ICA artifact correction Manual artifact rejection Inclusion of C3 or C4	–	(1) MP (2) Maximum ERD	[41]
	≤ 32	Frontal, central, parietal, occpitial	Active wet	256 Hz	(1) Time (2) Time-frequency	(1) Low-Pass [1 Hz] (2) Band-pass [6 - 32 Hz]	(1) Movement onset [− 3 s, − 2 s] (2) Movement onset [− 3 s, − 0.5 s]	–	(1) Dominant channel selection, matched filter (2) Welch’s method, channel/frequency pairs selection, classification (Naive Bayes) (1,2) Logistic regression of 1() and (2)	Movement intention	[40]
		Whole	Passive wet	500 Hz	Time-frequency	–	Movment onset [− 5 s, 18 s]	EOG-based artifact correction Manual trial rejection Baseline correction	(1) Band-pass filter [Dominant alpha frequency − 1 Hz/+ 1 Hz] (2) Band-pass filter [14.5 Hz, 24,5 Hz] Rectification	(1) Alpha-ERD (2) Beta-ERD	[39]
				250 Hz	Frequency	Band-pass [0.5 Hz–100 Hz]	–	Inclusion of central and front-central areas EOG-based artifact correction	–	Spatial distrubution of beta-band and mu-rythmn	[55]
	≤ 64	Whole	Active wet	5000 Hz	Time	Band-pass [0.05 Hz–2 Hz]	Force onset [− 2.5 s, 0.5 s]	Inclusion of frontal, central, and parietal areas Manual artifact rejection	–	MRCP	[47]
				100 Hz	Non-Linear	Band-pass [0.1–3 Hz]	–	EOG-based artifact correction Exclusion of peripheral channels	Standardize UKF decoder	Predicted joint angles	[48]
				1024 Hz	Time	High-pass [0.1 Hz]	Visual stimulus [− 0.4 s, 1.4 s]	Statistical channel rejection Manual artifact rejection ICA artifact rejection DIPFIT Baseline correction	Downsampling ERP microstate extraction Microstate selection LORETA	Microstate	[54]
			Active	1000 Hz	Non-Linear	Band-pass [0.1 Hz–3 Hz]	Walking stop/start (between 6 s to 6 min)	ASR Exclusion of peripheral channels Detrend	Standardize PCA UKF decoder	Predicted joint angles and velocity	[44]
				500 Hz	Time-frequency	Band-pass [1–40 Hz]	–	Manual channel correction Manual artifact rejection	SVD Morlet wavelet for each SVD topography Coefficient variation for delta, theta, alpha, and beta band	Resting-state SVD components for delta, theta, alpha and beta band	[52]
	≤ 128	Whole	Active wet	500 Hz	Frequency	–	Trail start [7.5 s, 17.5 s]	Manual channel rejection ICA artifact rejection	–	Band power	[49]
				1000 Hz	Time	High-pass [1 Hz] Low-pass [40 Hz]	Heel strike [− 0.5 s, 1.2 s]	Statistical trial/channel rejection	–	ERPs	[42]
			Passive wet	1024 Hz	Time-frequency	Notch-filter [50 Hz] Band-pass [0.5 Hz–200 Hz]	Movement onset [− 2 s, 4 s]	Manual artifact rejection Statistical channel rejection InfoMax artifact rejection	Co-registration of EEG sensors and anatomical landmarks Source estimation Morelet projections	Volume-based and intentsity-based lateralisation index	[53]
				5000 Hz	Time	Low-pass [45 Hz]	Visual stimulus [− 2.5 s, 2.5 s]	Manual trial/channel rejection Baseline correction Manual ICA artifact rejection	(1) Spatiotemporal representation vector, PCA (2) Standardize,FastICA	(1) Spatiotemporal vectors (2) ICA components mean and variance	[51]
			Wet	2048 Hz	(1) Frequency (2) Time-Frequency	Band-pass [2–200 Hz]	Movement onset [− 2 s, 3 s]	Statistcal channel rejection Manual trial rejection ICA artifact rejection	–	GED topography Time-frequency plot Theta z-score	[57]
fNIRS	8	Prefrontal, frontal	Optodes	–	Time	Band-pass [0.005 Hz–0.03 Hz]	–	Statistical channel/trial rejection Wavelet filtering Baseline correction	Correlation-based signal improvement Averaged HBo and HbR	Hdiff	[56]

#### Functional neuroimaging hardware

Among the technologies employed, EEG was the most commonly used (18 out of 19 studies)[39–57], while only one study used fNIRS [56].

The electrode number and placement varied significantly, ranging from single-channel EEG [45, 46] to low-density EEG [39–41, 43, 50, 55], common 64-channel EEG [44, 47, 48, 52, 54], and HD-EEG [42, 49, 51, 53, 57]. Most studies adhered to the 10–20 electrode placement system, except for five studies that used the 10–5 placement [49, 50, 53, 56, 57]. The selection of electrode placement varied according to the study’s cortical area of interest, such as prefrontal [45], central [46], frontal/central [43], prefrontal/frontal [56], frontal/central/parietal [50], and frontal/central/parietal/occipital [40]. Additionally, 5 out of 19 studies excluded electrodes during post-processing. Mazzoleni et al. [41], Liu et al. [55], and Colamarino et al. [47], Wasaka et al. [50] included only relevant electrode sites for feature extraction and Luu et al. [48], and Contreras-Vidal et al. [44] excluded known movement artifact prone peripheral channels. Luu et al. [48] further modified the 10–20 system due to the proximity of the GND and REF electrodes to the area of interest (motor cortex).

Active electrodes were the most commonly used in the studies, with 10 out of 19 employing them [40–44, 47–49, 52, 54]. However, not all studies reported whether wet or dry electrodes were used [43, 44, 50, 52]. Five studies used passive wet electrodes [39, 51, 53, 55, 57], one used dry electrodes [45], and one used dry foam electrodes [46]. Optodes were used for the fNIRS measurements [56].

The sample rate varied among studies, with most using sample rates of around 256 Hz [40, 43, 55], 500 Hz [39, 49, 50, 52], or higher frequencies of around 1000 Hz [42, 44, 54]. One study used a relatively low sample rate of around 100 Hz [48], and three used a very high sample rate of 2048 Hz [57] and 5000 Hz [47, 51]. In seven studies [44, 46, 47, 51–54], the recorded data stream was downsampled, particularly for studies using higher recording sampling rates of 500 Hz and above. Downsampling was done to 56 Hz [46], 128 Hz [44, 52, 54], 200 Hz [53], 1000 Hz [58], or 2048 Hz [47] during further signal preprocessing.

#### Functional neuroimaging data analysis

Out of 19 studies, eight studies were identified that used time-domain features [40–42, 47, 50, 51, 54, 56], five studies that used frequency-domain features [43, 46, 49, 55, 57], six studies that used time-frequency-domain features [39–41, 52, 53, 57], and two studies that used non-linear features[44, 48]. Three studies combined different domains as time and time-frequency domain features [40, 41] or frequency and time-frequency domain features [57].

The further description of the used functional neuroimaging methods is structured by their respective signal domain and the corresponding features, their extraction, and their domain and feature-specific preprocessing steps are described together. The description of the signal processing steps does not follow their exact order as applied in the publications, and instead, a standardized signal processing sequence is used.

##### Cortical time domain features

 For time-domain features, the studies mainly used event-related potentials such as induced gait perturbation-related potentials [42], movement-related cortical potentials (MRCP) [40, 41, 47, 50], or specific components of MRCP such as Bereitschafts potential (BP) amplitude [40], motor potentials (MP) amplitude [41]. Ibanez et al. [40] selected for the extraction of the BP the channel with the highest BP-Peak out of three virtual central channels and classified by a matched filter for movement intent. Colamarino et al. [47] used a Locality Sensitive Discriminant Analysis (LDSA) to classify movement intent based on the MRCP. Dipietro et al. [54] extracted microstates using a modified K-means algorithm based on submovements of movement correction during reaching, and Wasaka et al. [50] extracted spatiotemporal vectors and the mean and variance of ICA components. Liu et al. [56] extracted the index of hemoglobin differential (Hbdiff) based on the difference between de- and oxygenated hemoglobin levels from the fNIRS signal after correlation-based signal improvement.

##### Movement-related cortical potential

 The EEG signals were filtered at different frequencies during preprocessing. The publications targeting the extraction of movement-related cortical potential (MRCP) focused mainly on the lower frequencies up to around 1 Hz [40] to 2 Hz [47]. Wasaka et al. [50] included frequencies up to 20 Hz, and Mazzoleni et al. [41] included frequencies up to 40 Hz for the MCRP analysis, but additional extracted features of the time-frequency domain.

Only two studies on the MRCP stated the used re-referencing method, Laplacian re-referencing [40, 47]. The Laplacianan re-referencing method used by Ibanez et al. [40] was modified using the average of peripheral channels as a reference to minimize the individual weight of those.

For segmentation, the window size was chosen between − 5 s to − 2.5 s from movement/force onset to − 0.5 s to 1 s for the extraction of the MCRP.

Regarding artifact correction, only three studies extracting the MRCP [41, 47, 50] mentioned applying this preprocessing step. Two studies relied to some extent on a manual rejection of artifacts and the predetermined selection of electrodes for the area of interest (sensorimotor cortex) [41, 47]. Mazzoleni et al. [41, 50] study further adapted the included electrodes based on the laterality of the movement and applied ICA for artifact correction. Only Wasaka et al. [50] used non-manual statistical methods to reject artifact-prone trails.

##### Other cortical time domain features

 For the extraction of gait-related ERPs, a more comprehensive frequency range from 0–0 Hz was applied by Skidmore et al. [42], and subsequent artifact rejection was based on the statistical properties of the signal. Dipietro et al. [54] used quite an extensive frequency range from 0.1 to 128 Hz to extract microstates and applied manual channel and artifact rejection, and subsequently applied ICA and DIPFIT artifact rejection algorithms. McDermott et al. [51] used a frequency range of up to 45 Hz and manually rejected artifact-prone trials, channels, and ICA components. All studies used a common average filter (CAR) for re-referencing [42, 51, 54].

##### Cortical hemoglobin differential

 For the extraction of walking task-related Hbdiff, a band-pass filter at 0.005 Hz to 0.03 Hz was chosen. Liu et al. [56] further used fNIRS-specific artifact correction as channel and trial rejection based on the relative coefficient of variation for each wavelength and wavelet filtering of movement artifacts and final baseline correction.

##### Cortical frequency domain features

 Features of the frequency domain used in the included publications were theta/beta ratio [46], theta z-score [51], a matrix consisting of delta, theta, alpha, beta, and high gamma band for each electrode and trial [49], the spatial distribution of mu and beta band rhythms [55] and theta [57] over the cortex, and movement intent classification and tuning of kinematic parameters based on a support vector machine (SVM) classifier using the first and last component of Common Spatial Pattern (CSP) filter for the mu and beta band [43].

Only one study using frequency domain features stated using a filter for the range of 2–200 Hz [57]. Two studies noted the re-referencing of the EEG to Common Average Reference (CAR) [49, 57]. Three studies stated segmentation, either based on a moving window of 1 s [43] for online processing, a 5 s window around external balance perturbation onset [57], or a 10-s long window from 7.5 s after the trial start, within which the subject is expected to perform the given task [49]. All studies used artifact correction methods, such as manually excluding channels based on the area of interest [55] or due to artifacts in given channels [49] or trials [57]. Additionally, the studies used an algorithm for artifact correction based on either EOG recording [55], statistical parameters [57], or ICA-based artifact rejection [49, 57].

##### Cortical time-frequency domain features

 Time-frequency domain features such as event-related desynchronization (ERD) were extracted in multiple studies [39–41], with Platz et al. [39] focusing on the dominant alpha and beta bands, while the other publications considering a more extensive frequency range that encompasses multiple EEG bands [40, 41]. Pierella et al. [52] extracted topography maps of the first three SVD components, which accounted for 75% of the variance for each delta, theta, alpha, and beta frequency band using Morlet projections. Stokkermans et al. [57] extracted time-frequency maps around up to 40 Hz based on the generalized eigendecomposition (GED) component with the greatest eigenvalue. Steinisch et al. [53] extracted the volume-based and intensity-based lateralization index based on the oscillatory source power of neural clusters calculated using Morlet projections for intervals of 200 ms in the range of 0.5 to 30 Hz.

All studies using features of the time-frequency domain [39–41, 52, 53, 57] applied a filter, except for Platz et al. [39], which defined the frequency range later during feature extraction. Filters used in the studies ranged from 0.5 Hz [41, 53], 1 Hz [52], and 6 Hz [40] for the lower cutoff frequency to 32 Hz [40], 40 Hz [41, 52], and even up to 200 Hz in two studies [53, 57] for the upper cutoff frequency.

The most used re-referencing method (3 out of 6) was CAR [39, 52, 53]. Only one study used Laplacian re-referencing [40], and one did not state any re-referencing method [41, 57] for features of the time-frequency domain.

Regarding segmentation, most studies (4 out of 6) used movement onset as a reference for segmentation, and the window size ranged from − 5 s [39, 41], − 3 s [40], or − 2 s [53] to − 0.5 s [40], 1-s [41], 4-s [53], or even up to 18 s [39]. Stokkermans et al. [57] used the onset of external balance perturbation as a trigger and extracted cortical features in a window from − 2 to 3 s.

Mazzoleni et al. [41] applied the same artifact correction/rejection steps to extract their time-domain feature. Pierella et al. [52] used a purely manual approach for channel correction and artifact rejection. Both Platz et al. [39] and Steinisch et al. [53] used a combination of manual artifact rejection channel rejection based on the statistical features of the signal and the InfoMax algorithm [53] or manual trial rejection and additional electrooculogram-based (EOG) artifact rejection [39]. Stokkermans et al. [57] used the same artifact correction as for their frequency features stated above.

##### Cortical non-linear domain features

 Non-linear features of functional neuroimaging have been used to predict joint angle [48] and additional velocity [44] during walking. Both studies used the Unscented Kalman Filter (UKF) based on the standardized EEG signal to predict the aforementioned kinematic features.

To improve the quality of EEG signals, both studies used a frequency band of 0.1 Hz to 3 Hz [44, 48]. The frequency band was chosen to remove high-frequency noise and filter out artifacts that can contaminate the EEG signal. One study of the studies [44] also stated that they re-referenced the EEG based on CAR and used a segmentation window ranging from 6 s to 6 min, depending on the walking time of participants.

Both studies excluded peripheral channels to reduce the influence of movement artifacts on the EEG signal [44, 48]. However, there were differences in the artifact correction/rejection methods used. Luu et al. [48] used movement EOG-based eye artifact correction to remove eye movement artifacts, while Contreras-Vidal et al. [44] applied artifact subspace reconstruction (ASR) to correct for a wide range of artifacts, including eye blink, eye movement, and muscle activity.

### Fusion of functional neuroimaging and motion capture

The fusion of functional neuroimaging and motion capture has been utilized in various settings in the included studies. These settings are categorized as mentioned in the methods. Details on modality features are summarized in Table 7.

**Table 7:** Feature fusion and synchronization. Details on the synchronization and use of the multimodal feature. ‘–’ indicates that the authors gave no information on the respective matter

Category	Online/offline	Method	Trigger	Feature fusion details	References
Movement event detection	Offline	–	–	Movement event of left heel strike for calculation of EEG time domain features	[42]
		–	–	Movement event of toe-off for calculation of EEG frequency domain features	[55]
		Hardware	Pushbutton trigger	Movement onset of arm movement for calculation of EEG time-frequency features	[53]
		-	–	Grip force onset for calculation of EEG time domain features	[50]
	Online	Hardware	Photodiode/screen	Movement onset of hand for the training of a regression model based on EEG time domain features	[51]
Movement event detection; decoder training	Offline	Other	Digital signal	Training of naive Bayes classifier for detection of movement onset based on EEG time and time-frequency domain feautres	[40]
		–	–	Movement onset/offset of gait for training/evaluation of UKF decoding joint kinematics	[44]
Movement event detection; statistical relationship	Offline	Hardware	Trigger signal	Movement events of stepping behavior and leaning direction to model their relationship to EEG time-frequency domain feautres	[57]
		Hardware	Trigger signal	Movement onset of arm for calculation EEG time and time-frequency features Correlation of kinematic assessment scores and a kinematics and EEG time and time-frequency features	[41]
Decoder training	Offline	–	–-	Training/validation of a presonalized linear regression model prediciting motor perfomance index based on EEG frequency domain features	[49]
	Online	Software	Customized program	Training/validation of EEG UKF decoder that predicts joint kinematics	[48]
		Software	UDP	Control of robotic arm based on movement and movement initiation and tuning of kinematic parameters via EEG-based SVM classifier	[43]
		–	–	Training/evaluation of LSDA classifier predicting ankle movement onset based on EEG time domain features	[47]
Statistical relationship	Offline	Other	Manual	Training/Validation of a regression model for kinematic parameters and EEG time-frequency domain features	[39]
		–	–	Correlation between kinematics, muscle and brain activity	[52]
		–	–	Correlation of submovements onset/offset and EEG time domain feature	[54]
		–	–	Correlation between gait parameters and fNIRS time domain features	[56]
Attention	Online	–	–	Game interaction based on movement and feedback on attention level via EEG	[46]
		–	–	Game interaction based on movement and feedback on attention level via EEG	[45]

Nine of the 19 studies [40–42, 44, 50, 51, 53, 55, 57] fall under the category of movement event detection. Six of the 19 studies [40, 43, 44, 47–49] were categorized into decoder training. Six of the 19 studies [39, 41, 52, 54, 56, 57] evaluated the multimodal statistical relationship. Two of the 19 studies [45, 46] were categorized as parallel applications. Finally, four studies fell into multiple categories. For example, Ibanez et al. and Contreras-Vidal et al.[40, 44] utilized multimodal data for movement event detection and decoder training. In contrast, Mazzoleni et al. and Stokkermans at al. [41, 57] utilized the multimodal data for both movement event detection and statistical relationship.

#### Movement event detection

All studies used movement kinematics or kinetic to detect movement onset/offset or movement events during offline processing. Four of the nine studies used hardware synchronization [41, 51, 53, 57] using either a not closer defined push-button trigger [53], a trigger signal [41, 57] or indirectly via a photodiode on the screen [51]. One study only used a digital signal to synchronize the different modalities [40]. McDermott et al. [51] additionally correlated the movement signal with the EMG signal to ensure correct synchronization.

Movement event detection was either used to segment the EEG data based on specified movement events like heel strike [42] or toe off [55] during gait to calculate related EEG features based on the average of all related segments [42] or for separately for every single segment [55]. Three studies used general movement onset to calculate EEG features based on the average of all movement-related segments [41, 53] or to extract only cortical activity related to walking sequences during free walking by further considering movement offset [44]. Ibanez et al. [40] used the movement onsets to segment a training dataset for decoder training. Stokkermans et al. [57] used movement events to categorize the behavior-related EEG features for subsequent statistical analysis.

#### Decoder training

The included studies that used movement kinematics to train, evaluate, and update neural decoders did apply both online processing (3 out of 6) [43, 47, 48] as well as offline processing (3 out of 6) [40, 44, 49]. Four studies stated that the synchronization method used for offline modality fusion was either based on a not closer defined digital signal (see movement event detection [40]), manual synchronization, or in the case of online modality fusion using software-based synchronization based on a customized C++ program [48] or via network using UDP [43].

Contreras-Vidal et al. [44], and Luu et al. [48] used the recorded movement kinematics to train and evaluate a neural decoder based on UKF to predict intended joint angles and velocities (only [44]) from EEG for gait. Ibanez et al. [40] used a naïve Bayes classifier, and Colamarino et al. [47] various adaptive LSDA classifiers for the classification of movement intention of upper-limb reaching [40] or isometric ankle dorsiflexion [47] based on motion capture data. Frisoli et al. [43] classified movement intention for upper-limb reaching. However, they also used the output of the SVM classifier to tune the maximum joint jerk, acceleration, and speed. Mastakouri et al. [49] calculated normalized averaged jerk to measure motor performance based on movement kinematics and trained a transfer learning regression model to predict future personalized transcranial electrical stimulation (tES) training.

#### Statistical relationship

Studies exploring the statistical relationship between movement kinematics and neural activity did so only during offline processing [41, 52, 54, 56, 57]. One study only used a synchronization method based on a not closer defined trigger signal (see movement event detection) [41] and another study manually synchronized both modalities during post-processing [39].

Most studies (4 out of 5) calculated the correlation between biomechanical and neural parameters [41, 52, 54, 56]. Mazzoleni et al. [41], Pierella et al. [52], and Liu et al. [56] calculated the correlation between neural time and time-frequency features and kinematic parameters and calculated motor performance scores to investigate the cortical influence on motor performance. Platz et al. [39] applied a regression model to assess the relationship between kinematic parameters and EEG time-frequency features. As mentioned, Stokkermans et al. [57] used movement events (leaning direction and stepping behavior) to model the relationship between cortical dynamics and balance-related behavioral responses.

#### Parallel application

For this category of feature fusion, only two publications were found. In both, the authors did not state any use of synchronization method [45, 46]. Both studies used cortical changes related to attention to change exergame modalities controlled by a motion capture system [45, 46].

### Multimodal feedback

The participants were also provided feedback on their movements and cortical activity using different technologies. Ten of the included studies used a screen [41, 45–51, 53, 54] to present visual cues signaling the participants to move (1 out of 8) [47], moving a cursor towards a target on the screen based on movement input (4 out of 8) [41, 49, 51, 54], as interactive game incorporating feedback on movement (1 out of 8) [53], as feedback on the deviation from the required grip force [50], or both movement and cortical activity (3 out of 8) [45, 46, 48].

One study also provided assistive robotic feedback, if needed, in addition to visual feedback on a screen [52]. Two studies [43, 44] only provided robotic-assisted feedback during reaching based on eye-tracking, motion capture, and EEG [43], and gait based on the motion capture [44]. Ibanez et al. [40] used functional electrical stimulation (FES) triggered movement from the EEG-decoded movement intention as feedback. Skidmore et al. [42] changed the stiffness on the walking surface according to gait event parameters. Four studies provided no feedback on any modality [39, 55–57].

### Relevant study outcomes

As already demonstrated in the study characteristics, the different studies had different aims. Either the exploration of novel features based on combined neuroimaging and motion capture or the actual evaluation of applications for diagnostic, therapeutic, or assistive applications. Therefore a direct comparison of the study outcomes can not be made. In the following paragraph, the most relevant outcomes related to multimodal measurement in motor rehabilitation are in a semi-structured order.

#### Biomarkers

Five studies showed that multimodal data could help identify new neuromotor assessment and therapeutic approaches. For example, dual-tasking walking can promote motor cortex plasticity [56], surface stiffness manipulation can improve inter-limb coordination based on changes in the supraspinal neural circuitry [42], a griping task with visual feedback of the exerted force enhances ipsilesional movement-related cortical activity [50], and robot-mediated therapy can enhance cortical activation and upper limb motor performance in post-stroke subjects [41]. Whereas Stokkerman et al. [57] could find a statistical relationship between postural behavior responses and cortical dynamics, Platz et al. [39] were not able to find a statical relationship between EEG and kinematic parameters, such as acceleration and movement variation during a triangular finger movement.

#### Diagnostic applications

Four included studies concluded that multimodal neuromotor assessment could be a diagnostic tool to evaluate a patient’s state and progression. Topographic changes of submovement-related EEG microstates [54], combined metrics of EEG, EMG, and movement kinematics [52], or solely the combined assessment of EEG and movement kinematics [53] and novel gait training systems for the extraction of gait-related cortical activity [55], have been found to provide a more holistic evaluation of the rehabilitation progress and possible individualization of it.

#### Therapeutic applications

Five studies suggested new therapeutic applications based on combined neuroimaging and motion captures, such as treadmill training with hybrid BCI-controlled virtual avatar [48] on movement kinematics-trained neural decoders for robot-assisted gait training [44], a clinical pipeline for reaching movement intention classification [51] and feedback on attention levels during exergaming [45, 46]. These approaches can potentially improve neurological rehabilitation outcomes and be used for further assessment.

#### Assistive applications

Lastly, four studies explored the use of multimodal systems for developing assistive devices and their personalization. Examples include combining eye-tracking, EEG, and motion capture for object selection and tracking for reaching assistance [43], as well as training and calibrating neural decoders based on the individual movement jerk [49]. Personalized neural decoders and adaptive systems can help decode spontaneous movements and initiate timely assistance [40, 47].

## Discussion

This paper reviewed studies that have combined motion capture and functional neuroimaging within the context of motor rehabilitation. Given the novelty of this field, the emphasis was set on the technical implementation that facilitated such measurements. The following discussion highlights important considerations for selecting suitable hardware and signal-processing methods, and new methods that may facilitate future acquisition and analysis of multimodal data are pointed out. Moreover, we discuss the potential advantages of such multimodal measurements for motor rehabilitation, which can assist in the future development of novel rehabilitation applications.

### Current state of research

The characteristics of the extracted studies underscore the nascent stage of combining functional neuroimaging and motion capture for motor rehabilitation. The majority of included studies are relatively recent publications and primarily focused on basic or translational research. These studies explored novel movement-related cortical changes related to brain injuries during motor rehabilitation. They evaluated the feasibility of using multimodal biomarkers in future diagnostic and therapeutic applications. However, the lack of studies utilizing multimodal applications in clinical settings makes assessing their efficiency, reliability, and validity difficult.

Another indication of the field’s infancy is the extracted population characteristics. Small sample sizes, the predominant use of healthy participants, although the ultimate target groups were defined as patients, and the limited implementation of control groups support this observation. Furthermore, studies have mainly included participants suffering from a stroke, likely due to its high global prevalence and the need for improved rehabilitation approaches [61]. Nonetheless, other acquired brain injuries have a similar aetiology, suggesting that insight gained from stroke patients could be transferable [62].

The primary focus of the research has been on upper-body movements, such as reaching, possibly because around 80% of stroke survivors experience upper limb impairments, and these movements are well-researched in rehabilitation [63, 64]. This emphasis might also be attributed to the relevance of upper-body movements for DAIYLs and their relative simplicity of recording these movements (being relatively static) compared to lower-limb movements like gait.

### Motion capture in motor rehabilitation: current methods and applications

Most studies (13 studies) employed mechanical sensors, such as IMUs, force transducers, and goniometers, for biomechanical measurements. These sensors were attached directly to the body, handheld controllers, or integrated into wearable robotic devices. Mechanical sensors seem to be a popular and viable option for simple single-joint measurements, like movement event detection of a certain end-effector. While mechanical and magnetic sensors have a clinically acceptable spatial resolution (around 2$^{\circ }$ angular error for IMUs [65]) and are generally robust towards artifacts, external artifacts, like magnetic disturbances, gyroscopic drift, excessive motion speed [66], and shifting of the sensor itself [65], can introduce measurement errors. Additionally, incorrect application, inadequate initial calibration, and the underlying analysis algorithms can introduce further inaccuracies [65].

In terms of temporal accuracy, it was observed that mechanical sensors provide higher sample rates (> 2048 Hz) than marker-based (< 960 Hz) or markerless systems (< 200 Hz). Although high temporal resolution might not be crucial for acquiring slow movements encountered in rehabilitation, higher sample rates (> 1000 Hz) are beneficial for accurate synchronization between modalities, which in turn is necessary for the extraction of time- or phase-locked cortical potentials [67]. Still, not all included studies that used such features reported using any synchronization. Lower sample rates are sufficient for the extraction of solely biomechanical features, and the signals were often low-pass filtered during postprocessing, rejecting artifacts in higher frequencies.

Optical systems, including marker-based and markerless systems, were used to capture more complex movements involving multiple joints and limbs. Although considered the current gold standard, their use is typically restricted to laboratory environments due to size, cost, and complexity. A trend towards markerless systems was observed, which could be attributed to their easier setup, eliminating the need for precise sensors or marker placement [1]. Although marker-based systems are still considered the gold standard for motion capture, the necessary marker placement is sensitive to misplacement and calibration errors [68], skin movement artifacts [69], and marker occlusion artifacts. These errors can reduce the system’s theoretical sub-millimeter spatial accuracy [70], in some cases by up to 25 mm, translating to a joint angle error of approximately 10$^{\circ }$ [68]. This necessitates, in turn, more elaborate preprocessing, like marker labeling, gap-filling, and smoothing, before joint angle and rotation can be calculated with inverse kinematic models. However, only one study in the review reported implementing these steps for their marker-based recordings. Similar postprocessing steps should be used for IMU-based motion capture [71]. As the reviewed studies were primarily interested in voluntary movement events or kinematic parameters of end effectors, extensive preprocessing and biomechanical analysis was not required. Thus, careful task design can help mitigate some of these limitations and minimize the need for complex preprocessing.

In the majority of studies (11 studies), kinematic parameters were extracted, which are valuable for the automatic assessment of the rehabilitation process and therefore improved follow-up and individualization. Several other reviews identified biomechanical features such as range of motion, mean speed, mean distance, normalized path length, spectral arc length, number of peaks, task time metric, smoothness, and peak velocity as reliable features for movement assessment compared with traditional clinical assessments methods like the FMA [16, 64, 72]. Since hemiparetic movements show compensatory movement patterns and jerks and are slower, those are suitable biomechanical measures to identify functional changes [64].

Movement event markers, such as gait events or (sub)movement onset/offset, were also used in several studies (9 studies). This can facilitate the categorization of (online) data and enables the assessment of complex voluntary movements. These self-paced movements not only more accurately represent ecologically valid behavior but also exhibit distinct cortical dynamics compared to externally prompted movements [73, 74], potentially positively influencing motor rehabilitation outcomes [75].

Another observed application for kinematic metrics (e.g., joint angle and velocity) was to train and calibrate assistive systems (6 studies). The automatic acquisition of kinematic data helped with the individualization and adaptation of the, e.g., walking systems and, therefore, better acceptance and efficacy for future clinical applications.

### Functional neuroimaging in motor rehabilitation: current methods and applications

The analysis of the hardware used for functional neuroimaging systems indicates that EEG was the most utilized technology, while only one study employed fNIRS. As fNIRS is a relatively new technology, its utilization is naturally lower [76]. The advantage of fNIRS over EEG is its higher spatial resolution and simple application, but due to its reliance on the delayed hemodynamic response, it possesses a lower temporal resolution [76]. This delayed response could hinder its use in applications that rely on real-time feedback. As mentioned, a high acquisition frequency (> 500 Hz) is necessary to achieve correct synchronization between modalities and evaluate time- and phase-locked features. However, it is worth noting the majority of studies (9 studies) have primarily focused on slower cortical dynamics in the delta band range. Nonetheless, a few studies (2 studies) have examined movement-related cortical activities in higher frequency bands, such as alpha and beta ERD, and one study examined the mu rhythm.

Considering the electrode and optode placement, several studies (5 studies) naturally focused on the sensorimotor cortex, which is responsible for motor execution and sensory integration [77]. However, primarily motor initiation and execution are emphasized rather than sensory integration. Given the high prevalence of somatic deficits (up to 80%) in stroke survivors [78], elated cortical dynamics should be given more attention. In fact, recent research suggests investigating the widespread neural interactions throughout the entire brain, as nonmotor processes also influence motor behavior [79, 80]. Furthermore, sensor placement across the whole cortex enables extracting features in the source space. Although nearly all reviewed studies extracted the cortical features from the electrode space, signal analysis in source space can provide insight into the specific neural generators and disrupted functional connectivity between brain regions. However, the necessity of applying source space analysis depends on the application, and the associated increase in electrodes and its general complexity decreases usability in clinical applications [81].

Due to their sensitivity to signal artifacts, neuroimaging methods require several preprocessing steps. Those preprocessing steps depend on the features of interest and the underlying task. Artifact correction is one of the most critical preprocessing steps, especially for movement-related EEG measurements. Besides eye artifacts, muscle activity, cable, and electrode movement can introduce huge artifacts into the signal, which correlate spatially and temporally with the source [82]. This can be leveraged to correct movement artifacts based on the recorded movement activity [83, 84]. Findings in the present review show that manual artifact correction was predominant by offline visual inspection or statistical thresholds (11 studies), although this is generally seen as a time-intensive and somewhat subjective process [85]. Nine studies, thus, use additional semi-automatic or solely automatic artifact correction methods, like blind source separation. Those algorithms are commonly based on subtracting components related to predefined artifact templates from the signal. Examples from the included studies are DIPFIT and online-processing capable algorithms like InfoMax and artifact subject reconstruction (ASR). ASR and its variation Riemannian ASR (rASR), as well as the AMICA algorithm, perform favorably for MoBI tasks but with the limitation that they require an artifact-free baseline and the manual setting of hyperparameters [85, 86]. On the other hand, a study by [87] suggests that movement artifacts are barely noticeable in the signal for slow walking and weak peripheral movements, and artifact correction is not required.

Several studies (4 studies) used movement-related time-domain features like the well-studied MRCP and its components (e.g., BP). Such event-related potentials (ERPs) are time- and phase-locked features, and, therefore, good synchronization of the different modalities for extraction of movement events is essential, specifically for online applications [67]. However, none of the included studies explored phase changes during movement execution, such as phase locking or synchrony, which correlate with the FMA, as shown in several studies summarized by Milani et al. [88]. For motor rehabilitation, the early components of the MRCP are frequently used to extract information on premovement cortical processes such as movement intention. This was used to initiate FES or assistive robotics movements and, thereby, closing the cortico-peripheral feedback loop, facilitating neuroplastic changes. However, MRCPs were also used to assess the changes in subcortical and cortical reorganization, which can aid in predicting the outcome of motor recovery. Besides classic parameters, like their latency and amplitude, their interhemispheric distribution was used as an indicator for motor recovery. The included studies analyzed this spatial distribution of the time domain using microstates. So far, MRCPs were mainly acquired for single-joint movements in healthy participants unrelated to complex real-world movements. Olsen et al. [89] concluded that the lack of ecological validity, mainly caused by technical difficulties, might limit the relevance of MRCPs for clinical applications.

Features of the time-frequency domains are time-locked to a particular event. Like the MRCP, ERD and event-related synchronization (ERS) are well-study movement-related features, too. ERS in the delta and theta bands are also known cortical features related to movement [90], but were not used in any included study. These features were used for the assessment of stroke and their use for assistive applications, similar to MRCPs. Motor-related changes were often observed in the mu and alpha bands and seem highly reliable measures [16, 91]. However, only changes in the observed changes in lower frequency bands, like the delta and theta bands, were connected to motor recovery by Seas et al. [92]. The spatial distribution of time-frequency features, like ERD/ERS lateralization or asymmetry index, can be used to support correct interhemispherical activation patterns, as the interhemispherical balance is altered toward greater activation of the ipsilesional hemisphere [88, 91]. However, this seems to be debated and rather dependent on the individual pathology and task design [93].

The unscented Kalman filter seems to be a popular choice for decoding gait joint kinematics in real-time. If precise control is needed, Nakagome et al. [94] suggested using recurrent neural networks, like gate recurrent units or quasi-recurrent neural networks. However, their real-time applicability has to be evaluated closer first.

### Multimodal signal synchronisation

As mentioned, not all authors stated their used methods of synchronization nor potential synchronization latencies or jitter. In some cases, this was not required, as the features were calculated independently of each other for a whole session and then aligned offline. However, good temporal synchronization between the various features is essential for online applications and time- and phase-locked movement-related features to acquire meaningful results [67]. This can necessitate higher sample rates to extract such cortical dynamics, although low sample rates are sufficient for biomechanical analysis of the relatively slow movements observed in our review (< 12 Hz) and clinical monitoring (< 20 Hz) [95]. The mismatch between sample rates can, if not correctly synchronized, introduce an uncertainty of several milliseconds, which can be problematic for online applications of time- and specifically phase-locked features [67]. Based on the time constraints of EEG/EMG connectivity measurements, Artoni et al. [96] recommend that the maximum misalignment between modalities should stay below 10 ms. Yet, this can be relaxed for other features, e.g., calculated over larger windows/segments.

Hardware-based synchronization based on TTL triggers is still considered the gold standard and gives high temporal accuracy with low latencies and jitter. However, this requires specialized equipment supporting hardware synchronization, which is often not economical or technically possible. Therefore, software synchronization is a simple alternative. Using protocols like LabStreamingLayer (LSL), various systems can be synchronized via a local network [97]. The temporal accuracy is sufficient for most applications if the hardware latencies and eventual jitters are measured and validated first [96].

### Modality fusion of functional neuroimaging and motion capture in motor rehabilitation

Previous reviews on modality fusion have emphasized the benefits of utilizing either functional neuroimaging or biomechanical analysis as unimodal measurements [16, 20, 21]. Similarly, the combination of functional neuroimaging and electrophysiological measurements has been explored in multimodal approaches [32, 33]. However, integrating biomechanical features into this multimodal analysis has received comparatively less attention. To bridge this gap and explore the synergistic benefits of combining motion capture and functional neuroimaging, this section presents its current state of research.

Multimodal data fusion can be defined as a technique that aims at the synergistic integration of different data sources to extract information that may not be accessible through a single source. Not unlike in nature, where humans and animals assess situations and make decisions based on the analyzed information of multiple senses. However, multimodal data fusion should not only focus on using sensor data to *complement* the overall information by leveraging the specific strengths of given modalities or *reveal* new information unavailable to unimodal measurement, but also *constrain* each other for more reliable outputs [98]. In the case of fusing biomechanical and cortical features, motion capture can be primarily used to provide contextual and behavioral information, providing a more comprehensive and holistic understanding of the related neuromotor processes in ecologically valid environments. Additionally, such multimodal data allows for more robust and reliable information in disambiguous situations common in clinical applications (e.g., due to acquisition errors, environmental noise, and individual variability or complex behavioral tasks).

The extracted publications confirm the mentioned benefits of fusing neurophysiological and biomechanical features for motor rehabilitation. The majority of these benefits were of a technical nature, as highlighted in 13 studies. These technical benefits include improved extraction of movement event markers or the online training of neural decoders based on acquired movement kinematics. The possibility of extracting cortical dynamics related to voluntary movements is of particular value. These self-paced movements represent not only ecologically valid behavior more accurately but also exhibit distinct cortical dynamics compared to externally prompted movements [73, 74], potentially influencing motor rehabilitation outcomes [75]. It is suggested that the supplementary motor area and subcortical areas are involved predominantly in self-initiated voluntary movements, whereas the premotor cortex has a higher involvement in externally cued movements [99]. Depending on the individual’s lesion site, rehabilitation training in ecologically valid scenarios requiring voluntary movement initiation might be beneficial [99]. Furthermore, by using movement kinematics for decoder training, the individual adaptation of especially assistive and therapeutic devices, like robot-assisted training, is facilitated and accelerated. For future clinical applications, easy usability and accurate decoding are necessary to achieve broad adoption of new rehabilitation technologies. This, however, presupposes that the additional hardware does not add any additional difficulties. Noteworthily, two studies differed by not assessing movement-related cortical processes but instead measured attention levels to improve user experience during training. Although this feature is not directly related to movement, as mentioned, monitoring nonmotor processes, e.g., attention levels, might benefit motor learning by adapting task complexity and aiding the generalizability to real-world situations [4, 100].

Another commonly observed fusion technique was the use of the statistical relationships between biomechanical and cortical features to assess the patient’s current state and predict possible rehabilitation outcomes. Combining biomechanical analysis to gain information on the quality of movement execution and synchronous evaluation of cortical changes during movement planning and initiation enables a holistic insight and increases reliability [52]. Therefore, more accurate patient progress predictions can be made, and the rehabilitation plan can be adjusted appropriately [16]. The reviewed studies, however, had different study designs and extracted different biomechanical features, which does not allow for any generalization. It should be further noted that included studies only analyzed linear statistical relationships (e.g., Pearson correlation). However, the reviewed studies decoding movement kinematics suggest using nonlinear filters, such as the UKF, to account for the nonlinear relationship between cortical dynamics and movement kinematics. Yang et al. [101] furthermore suggested considering the complex nonlinear properties of the sensorimotor control loop, specifically for proprioceptive afferent feedback.

To improve cortical-peripheral coupling, therapeutic applications should further consider the right choice of feedback. Feedback on movement-related changes in cortical dynamics is a common BCI application used to restore interrupted cortico-peripheral coupling and thus improve functional outcomes [4]. In this review, only a few studies (6 studies) gave feedback on changes in cortical dynamics, like movement intention, whereas most studies gave feedback solely on movement. Considering the overall limited research on the fusion of biomechanical and cortical features, it is reasonable that the incorporation of appropriate feedback may not have been a primary focus. Further studies may be necessary to address this gap and systematically develop effective feedback designs for this area of research.

In terms of clinical validity and reliability, current combined measures’ are not yet proven sufficiently, thus hindering the implementation of clinical studies [16, 102]. Further, considering the inter-subject variability of motor disabilities and their cortical manifestations, evaluating those measures required large clinical trials, which have not been done so far [34]. However, several authors argue that the use of multimodal biomarkers could increase robustness for clinical applications. Either due to the elimination of time-intensive user-specific adaptation of underlying classification algorithms [103] or the increased reliability and validity of the fused features due to the inherent redundancy of multimodal systems [98].

From a technological standpoint, the recent advancements in the related fields have made clinical applications of fusion-based applications more feasible. Notably, the continuously improving comprehensive spatial depiction and high spatiotemporal accuracy of some motion capture allow for novel clinical applications. This presents a distinct advantage of motion capture when compared to other movement-related modalities, such as the commonly used EMG. However, it should be taken into consideration that compared to EMG, motion capture is less sensitive to residual muscle movements and thus also to involuntary muscle activity due to, e.g., spasticity post-stroke [104]. On the other hand, motion capture technologies can measure passive movements and thus be used for the assessment of cortico-peripheral coupling even when no residual muscle activity is present. Furthermore, continuous technological advances in neuroimaging enable new clinical applications, as well. Notably, due to novel electrodes/optodes technologies [105], the miniaturization of mobile brain imaging hardware, and improved artifact rejection algorithms [106] will most likely increase the clinical usability of functional neuroimaging in the coming years.

In summary, it may be asserted that the implications for motor rehabilitation of multimodal measurements based on motion capture and functional neuroimaging are not entirely predictable. Current studies mainly evaluate the technological feasibility and build a basic understanding of cortical changes post-stroke and during rehabilitation training. The fusion of both modalities has been shown to aid in a better understanding of cortical changes’ effect on functional improvement for ecological valid tasks. Based on those insights, objective and robust multimodal assessment methods, considering the mechanism of cortical-peripheral coupling, might aid in better planning of rehabilitation measures and even promote new rehabilitation technologies, as suggested by multiple other publications [6, 16, 20, 34].

### Suggestions for future research

Development in related fields might aid in overcoming the mentioned shortcomings, which could facilitate the use of combined motion capture and functional neuroimaging in future rehabilitation applications and research.

As mentioned, a trend toward markerless motion capture was observed. AI-based motion capture systems, such as camera-based human pose estimation (HPE), offer an alternative to traditional systems for tracking multiple persons’ whole-body kinematics in diverse environments without the intrusive placement of sensors or markers and at a low cost [107]. The Kinect or Leap Motion Orion, included in the review, are examples of AI-based systems. Whereas the Kinect and Leap Motion Orion are based on proprietary hardware and software, are both open-source HPE pipelines available [108], as well as commercial solutions (Theia 3D). The significant progress in spatial and temporal accuracy and robustness over the last few years [107] could enable clinical applications. Current offline systems show acceptable spatial accuracy of < 4$^{\circ }$ angular errors, while online HPE is approaching the maximum permissible limit for clinical applications of 5$^{\circ }$ angular errors [108, 109]. Temporal accuracy depends on the camera system (consumer-grade webcams: 30–60 Hz, specialized high-framerate USB cameras:   750 Hz) and for online applications on the employed HPE algorithm. However, despite the potential clinical benefits, challenges remain unresolved, such as the requirement for camera calibration, occlusion handling, high computational hardware demands [108], and lacking knowledge of the technology’s reliability and validity. It should be noted that most HPE algorithms are trained on manually annotated or marker-based motion capture data, which, as discussed above, can introduce errors.

Current feature fusion did not take the intercortical and peripheral coupling fully into account. However, there is a general shift from analyzing different cortical areas independently to focusing on their interconnectivity [80, 110]. Going even further and studying changes in the long-range top-down (efferent) and bottom-up (afferent) cortico-peripheral coupling could aid in understanding the role of the motor feedback loop during rehabilitation [34]. A common feature for measuring efferent cortical-peripheral coupling is cortico-muscular coherence (CMC), based on measurements of muscle activity and underlying cortical activity [32, 111]. Recent studies even indicated its potential use in a BCI-based rehabilitative approach. By utilizing CMC in combination with neuromuscular electrical simulation Guo et al. [103] were able to improve voluntary wrist movement post-stroke and simultaneously suppress maladaptive, compensatory strategies. They argued that motor improvements could be attributed to neuroplastic changes in the afferent and efferent corticospinal tracts.

More recently, the afferent sensory feedback features have been investigated, as somatic deficits have a high prevalence (up to 80%) in stroke survivors [78]. Cortico-kinematic coherence (CKC) evaluates the functional connection between cortical activity and movement kinematics and mainly reflects proprioceptive feedback to the primary sensorimotor cortex [112]. Thereby it could help to asses disruption in cortical somatosensory processing based on the correlating topographic changes in cortical activity [34]. CKC seems to be a robust feature with potential for clinical rehabilitation; however, it was not used in this specific context [113]. The use of deep-learning-based motion capture to calculate CKC was successfully demonstrated by Maezawa et al. [114] to map cortical areas involved in tongue movement. Another feature that can assist in evaluating somatosensory impairments based on the coherence between EEG and perturbed mechanical movement is position-cortical coherence (PCC). PCC, however, requires a more elaborate setup compared to CKC but has shown to be a reliable test for afferent pathway integrity in stroke survivors [115]. As the named futures are currently mainly analyzed on their linear relationships between the modalities, Bao et al. [34] suggest analyzing non-linear relationships in the feature, better representing the somatosensory control loop.

Finally, current reviews on multimodal measurements for clinical applications [32, 33], alongside this present work, predominantly focus on the fusion of two modalities. Only Maura et al. [16] identified a singular study where multiple modalities were combined (specifically, EEG, EMG, and kinematic measurements). The current emphasis on bi-modal measurements undoubtedly represents an important first step in comprehending this emerging methodological approach. However, a comprehensive exploration of the fusion of multiple additional modalities, weighing of their respective advantages, disadvantages, and usability, might provide additional insight into cortical processes related to various behavioral responses.

## Conclusion

In our review article, we explore the fusion of functional neuroimaging and motion capture techniques in the context of motor rehabilitation in order to understand the diversity and maturity of technological solutions employed and explore the clinical advantages of this multimodal approach. We investigate the technological advancements that have facilitated the synchronous acquisition and analysis of complex signal streams, encompassing neurophysiological data (such as EEG and fNIRS) and behavioral data (such as motion capture).

Despite being in an early stage of research, the combination of motion capture and functional neuroimaging shows promising technical and therapeutic benefits for motor rehabilitation in terms of both clinical assessment and therapy.

However, the field still relies on traditional methods of data acquisition and analysis methods, which stay behind the current technological possibilities. Similarly, the final fusion of modalities relied on simple methods, such as exploring linear statistical relationships and voluntary movement-related localized cortical dynamics. Additionally, the synchronization of data streams was underreported. The identified technical benefits helped to facilitate assessing cognitive processes in an ecologically available environment and improve the usability and robustness of rehabilitative approaches. From a therapeutic perspective, this further enabled a holistic understanding of cortico-peripheral coupling and possibly opened new avenues for developing novel neuro-rehabilitation methods. Future research should explore the possibility of evaluating somatosensory processes that could allow for personalized proprioceptive training and novel rehabilitation routines. Furthermore, incorporating current technological advances in hardware and signal processing is essential for enhancing usability and complex multimodal data analysis.

Addressing these challenges is essential for advancing the development and eventual clinical application of combined motion capture and functional neuroimaging approaches, which can provide insights into cortical mechanisms during movement, guide rehabilitation practices, and serve as a tool for assessment and therapy in neurorehabilitation.

## Data Availability

Not applicable.

## Abbreviations

ADLs: Activities of daily living
AMICA: Adaptive mixture independent component analysis
ASR: Artifact subspace reconstruction
BCI: Brain-computer interface
BP: Bereitschaftspotential
CAR: Common average reference
CKC: Cortico-kinematic coherence
CMC: Cortico-muscular coherence
CSP: Common spatial pattern
EEG: Electroencephalography
EMG: Electromyography
EOG: Electrooculogram
ERD: Event-related desynchronisation
ERS: Event-related synchronisation
FES: Functional electrical stimulation
FMA: Fugl-Meyer Assessment
fMRI: Functional magnetic resonance imaging
fNIRS: Functional near-infrared spectroscopy
GED: Generalized eigendecomposition
GND: Ground
Hbdiff: Cerebral hemoglobin oxygenation Oxygenated minus deoxygenated hemoglobin
HD-EEG: High-density-electroencephalography
HPE: Human pose estimation
ICA: Independent Component Analysis
IMU: Interial measurement unity
LDSA: Locality Sensitive Discriminant Analysis
LSL: LabStreamingLayer
MEG: Magnetoencephalography
MoBi: Mobile Brain/Body Imaging
MP: Motor potential
MRCP: Movement-related cortical potential
PCC: Position-cortical coherence
PET: Positron emission tomography
PRISMA: Preferred reporting items for systematic reviews and meta-analyses
rASR: Riemannian artifact subspace reconstruction
REF: Reference
RGB: Red, green, blue
RQ: Research question
SD: Standard deviation
SVD: Singular value decomposition
SVM: Support vector machine
tES: Transcranial electrical stimulation
TBI: Traumatic brain injury
TOF: Time-of-Flight
TTL: Transistor-Transistor-Logik
UDP: User data protocol
UKF: Unscented Kalman filter
